# Two-Step Triethylamine-Based Synthesis of MgO Nanoparticles and Their Antibacterial Effect against Pathogenic Bacteria

**DOI:** 10.3390/nano11020410

**Published:** 2021-02-05

**Authors:** Ramiro Muñiz Diaz, Pablo Eduardo Cardoso-Avila, José Antonio Pérez Tavares, Rita Patakfalvi, Virginia Villa Cruz, Héctor Pérez Ladrón de Guevara, Oscar Gutiérrez Coronado, Ramón Ignacio Arteaga Garibay, Quetzalcoatl Enrique Saavedra Arroyo, Virginia Francisca Marañón-Ruiz, Jesús Castañeda Contreras

**Affiliations:** 1 Centro Universitario de los Lagos, Universidad de Guadalajara, Lagos de Moreno 47460, Mexico; ramiromunizdiaz@gmail.com (R.M.D.); ptavaresjose@gmail.com (J.A.P.T.); virginia.villa@academicos.udg.mx (V.V.C.); hperez@culagos.udg.mx (H.P.L.d.G.); ogutierrez@culagos.udg.mx (O.G.C.); vmaranon@culagos.udg.mx (V.F.M.-R.); jes@culagos.udg.mx (J.C.C.); 2 Centro de Investigaciones en Óptica, A.C, León 37150, Mexico; pecardosoa@hotmail.com; 3 Centro Nacional de Recursos Genéticos, Instituto Nacional de Investigación Forestal, Agrícola y Pecuaria, Tepatitlán de Morelos 47600, Mexico; arteaga.ramon@inifap.gob.mx; 4 Instituto Tecnológico Superior de Irapuato, Irapuato 36821, Mexico; ensaavedra@itesi.edu.mx

**Keywords:** magnesium oxide nanoparticles, triethylamine, solvothermal, antibacterial activity, reactive oxygen species

## Abstract

Magnesium oxide nanoparticles (MgO NPs) were obtained by the calcination of precursor microparticles (PM) synthesized by a novel triethylamine-based precipitation method. Scanning electron microscopy (SEM) revealed a mean size of 120 nm for the MgO NPs. The results of the characterizations for MgO NPs support the suggestion that our material has the capacity to attack, and have an antibacterial effect against, Gram-negative and Gram-positive bacteria strains. The ability of the MgO NPs to produce reactive oxygen species (ROS), such as superoxide anion radicals ($O_{2}^{•-}$) or hydrogen peroxide (H_2_O_2_), was demonstrated by the corresponding quantitative assays. The MgO antibacterial activity was evaluated against Gram-positive *Staphylococcus aureus* and Gram-negative *Escherichia coli* bacteria, with minimum inhibitory concentrations (MICs) of 250 and 500 ppm on the microdilution assays, respectively. Structural changes in the bacteria, such as membrane collapse; surface changes, such as vesicular formation; and changes in the longitudinal and horizontal sizes, as well as the circumference, were observed using atomic force microscopy (AFM). The lipidic peroxidation of the bacterial membranes was quantified, and finally, a bactericidal mechanism for the MgO NPs was also proposed.

## 1. Introduction

Biological pathogens continue to attract attention in the health sector. To solve this problem, it is highly necessary to develop effective antimicrobial agents to control bacterial populations [1,2,3]. Generally, antibacterial agents can be categorized as organic or inorganic antibacterial agents. Organic antibacterial agents have been widely studied. However, they have shortcomings, such as a low resistance to processing conditions, which limit their applications [4]. As a result, inorganic antibacterial agents have attracted much interest in bacterial control [5]. The main advantages of inorganic antibacterial agents are the improved stability under harsh processing conditions; therefore, the use of nanostructured oxide materials has become an option against these pathogens [4,5].

These oxide nanomaterials have flourished in various biological areas [6,7,8,9] and have been widely studied due to their interesting physical and chemical properties. Oxide nanomaterials have also been shown to be safe for humans and animals [10,11,12]; however, before their widespread use is approved, their mechanisms of action need to be understood to elucidate and predict the potential ecotoxicity and environment impact. In general, inorganic nanomaterials (i.e., TiO_2_, CuO, ZnO, MgO, CaO, Al_2_O_3_, SiO_2_, Fe_2_O_3_, and CeO_2_) are used in decontamination against biological pathogens and as agents for cancer treatments, among others [9,13,14,15].

The toxicity of these materials is associated with their superficial oxygen [16,17], and, due to point defects on their surface, they can become very reactive materials. These punctual defects can generate holes ($h^{+}$) and electrons ($e^{-}$), under illumination or even in the absence of light depending on the metallic oxide [17,18,19], promoting the production of reactive oxygen species (ROS), such as hydroxyl radicals $\left({\mathrm{OH}}^{•}\right)$, superoxide anion radicals ($O_{2}^{•-}$), hydrogen peroxide ($H_{2}O_{2}$), and perhydroxyl radicals $\left({\mathrm{HO}}_{2}^{•}\right)$ [17,18,20]. These species are the principal mediators to damage the active components that maintain the physiological and morphological functions of the microorganisms.

The ROS can attack the external and internal organic material of the pathogenic microorganisms; an example of this is the oxidative degradation of lipids, where the main initiators are ${\mathrm{OH}}^{•}$, and the results are the loss of homeostasis of the cell [21,22,23,24]. In addition, alternative mechanisms for the release of metal ions have been proposed in the literature [25,26]. Even though many bactericidal mechanisms are not fully understood, there are several premises to efficiently deactivate these microorganisms. The oxide nanomaterials must have many superficial punctual defects and a strong positive charge that will allow the material close interaction with the negatively charged bacterial surface [9].

The diversifying of methods for producing metallic oxides nanoparticles has enabled these materials to be applied among different areas, including catalysis, ceramics, reflecting and anti-reflecting coatings, sensors, and chemical residue remediation [10,27,28,29,30,31,32]. Among the well-known metallic oxide nanoparticles (TiO_2_, CuO, ZnO, etc.), magnesium oxide (MgO) is the least studied for biological and antimicrobial applications [9]; nevertheless, its high ionic character, simple stoichiometry, crystalline structure, the presence of superficial punctual defects, the availability of materials, their low cost, and their excellent biocompatibility make the magnesium oxide nanoparticles (MgO NPs) an excellent candidate for biological applications [21,22,27,29,33,34,35,36,37,38,39].

In contrast to TiO_2_, magnesium oxide does not require ultra-violet (UV) light activation to present antibacterial effects [18,21,22,36,37,40]. Even when small Mg^2+^ ions can be liberated, their toxicity is negligible compared to the toxicity associated with the Zn^2+^ ions liberated from ZnO NPs [25,41]. Many MgO nanoparticle (NP) synthesis methods have been developed with a high surface area and controlled morphology [11,19,42]; but the solvothermal method is very convenient due to the low temperatures of reaction, its simplicity, low cost, and high yield [43,44,45].

The reported methods for the production of MgO NPs use different precipitating agents, such as sodium hydroxide (NaOH), tetrapropylammonium hydroxide (TPAOH), or sodium borohydride (NaBH_4_), among others, and protecting agents such as organic species (polyols), surfactants, or polymers (long-chain amines) [27,46,47]. Triethylamine (TEA), a weak base, has been used in the production of ZnO NPs [48], and inspired by this work, we present a novel synthesis of MgO NPs based in the precipitation of magnesium acetate by TEA. Our work also presents the physical and chemical characterization of the MgO NPs, and the evaluation of the antibacterial effect against *Escherichia coli* and *Staphylococcus aureus* bacteria, as well as the morphological changes induced by MgO NPs through atomic force microscopy. Finally, a bactericidal mechanism for the MgO NPs is proposed.

## 2. Materials and Methods

Magnesium acetate tetrahydrate (≥99%), polyethyleneimine (PEI, 50% in water, 750 kDa), triethylamine (TEA, ≥99%), nitro blue tetrazolium (NBT, ≥90), and resazurin sodium salt were purchased from Sigma-Aldrich Chemical Co. St. Louis, MO, USA; a TBARS (Thiobarbituric acid reactive substances) parameter assay kit (KGE013; R&D Systems, Minneapolis, MN, USA), soluble starch (Merck Millipore, Burlington, MA, USA), sodium chloride (≥99%, Karal, León, Mexico), potassium iodide (≥99%, Jalmek, San Nicolás de los Garza, Mexico), hydrochloric acid (36–38% Jalmek, San Nicolás de los Garza, Mexico), and hydrogen peroxide (Karal, León, Mexico) were used as received.

Thermogravimetric analysis (TGA) was carried out on Q600 apparatus (TA instruments, New Castle, DE, USA) from 25 to 1000 °C using a 10 °C/min ramp under an extra-dry air atmosphere. Scanning electronic microscopy (SEM) was performed using a JSM-7800F JEOL (Tokyo, Japan) microscope: the powder samples were placed on top of graphite tape and the images were obtained using a 1 kV accelerating voltage. Open-source software ImageJ was used to analyze the SEM images to calculate the mean size of the PM and MgO NPs; at least 200 particles were measured in their longest dimension. X-ray diffraction patterns were obtained from 10 to 80 degrees using a D2 Phaser X-ray diffractometer equipped with a Cu kα (λ = 0.154 nm) radiation source (Bruker Corporation, Billerica, MA, USA).

UV–Vis absorbance spectra were acquired by a Cary 60 spectrometer (Agilent Technologies, Santa Clara, CA, USA) in the region from 200 to 1100 nm using a 10 mm quartz cell. Fourier-transform infrared spectroscopic analysis was performed in the region from 4000 to 500 cm^−1^, where the sample was placed on a KBr matrix and were pressed to form a translucent pellet or thin films (Frontier model, Perkin Elmer, Waltham, MA, USA). Atomic force microscopy (Nanosurf easyScan 2, Liestal, Switzerland) was used to observe the morphological changes the MgO NPs induced in the bacteria. A total of 250 µL of the microdilution samples were washed by adding 750 µL of deionized water (DIW) and centrifuged at 2000× *g* for 5 min at 5 °C. The precipitates were washed three more times by centrifugation by adding 1 mL of DIW. Finally, 20 µL of the final suspension was allowed to dry for 1 h on top of an ultrasonically cleaned microscope glass slide.

Solvothermal synthesis of MgO nanoparticles: for the synthesis of MgO nanoparticles, 1.715 g (2.5 × 10^−3^ mol) of magnesium acetate tetrahydrate was dissolved by sonication in 32.00 mL of a 3% polyethyleneimine (PEI) methanolic solution in a 50.00 mL Ace pressure tube (Sigma-Aldrich Chemical Co. St. Louis, MO, USA). When the magnesium salt solution was clear, it was magnetically stirred, and 5.575 mL of triethylamine (TEA) was added dropwise. The glass vial was closed and heated for 3 h in an oil bath at 120 °C. The product was left to age for 24 h, after which it was washed by centrifugation at 4000× *g* for 15 min. The precipitate was dispersed in methanol, and this washing process was repeated seven more times to remove the residual organic compounds. Next, the product was dried at 70 °C for 24 h and ground in a mortar to obtain precursor microparticles (PMs). Finally, these PMs were calcinated at 800 °C for 5 h to obtain the MgO nanoparticles. We supposed that PMs are mainly composed of anhydrous magnesium acetate particles covered by PEI [49].

Superoxide anion radical $O_{2}^{•-}$ quantification: superoxide anion radicals were quantified by monitoring the degradation of nitro blue tetrazolium (NBT) by UV–Vis absorption spectroscopy. In the presence of $O_{2}^{•-}$, NBT degrades to formazan, which is not soluble in water, and thus the absorption band at 259 nm associated with NBT will decrease. The test was performed using 20 mL of a 2.5 × 10^−4^ M NBT in a NaCl 0.9% m/v solution to which 5 mg of MgO NPs were added. Two different lighting conditions were analyzed for the generation of $O_{2}^{•-}$; the first sample was kept in the dark and at 43 °C, while the second one was illuminated by a 365 nm UV lamp for 2 h (Blak-Ray™ B-100AP High-Intensity lamp, UVP LLC, Upland, CA, USA). The lamp elevated the temperature of the sample to 43 °C, and for this reason, we decided to heat the other sample with a hot plate to carry the experiments under the same thermodynamic conditions. For both samples, an aliquot of 3 mL was extracted every 20 min, and these aliquots were centrifugated at 4000× *g* for 20 min. The supernatants were then analyzed by UV–Vis spectroscopy. By using the stoichiometric relationship in the NBT reduction to formazan reaction, the superoxide production was calculated [20,50,51].

Hydrogen peroxide quantification: the hydrogen peroxide production by the MgO NPs was quantified by the iodometry technique through monitoring the oxidation of the iodide ion to iodine with a sensing probe that changed in color with this reaction. Iodine and starch form a blue color complex that can be measured by UV–Vis spectroscopy, and by the construction of a calibration curve, the $H_{2}O_{2}$ produced by the MgO NPs could be calculated. Three samples were prepared by dissolving 100 mg of MgO NPs in 20 mL of DIW by sonication in the dark for 20 min. Next, two different light conditions were used: 365 nm UV irradiation for 4 h and a sample kept in the dark and heated at 43 °C with a hot plate.

After 4 h of magnetic agitation under these conditions, the samples were centrifuged at 4000× *g* for 20 min, and 5 mL of each supernatant was transferred to a 10 mL volumetric flask. Next, 500 µL of NaCl solution (200 mg/mL), 200 µL of HCl solution (3.6% *v*/*v* in water), 300 µL of starch solution (10 mg/mL), 300 µL KI (10 mg/mL), and finally DIW was added to bring the mix to 10 mL. The solution was sonicated for 20 min and the UV–Vis spectra were obtained. With the use of a calibration curve that was constructed for $H_{2}O_{2}$ concentrations from 0 to 5 μg/g, the $H_{2}O_{2}$ produced by the MgO NPs was directly calculated using the absorbance value at 545 nm [52].

Antimicrobial activity evaluation: *Escherichia coli* (ATCC 25922) and *Staphylococcus aureus* (ATCC 25923) are microorganisms with high pathogenicity. These microorganisms were cultivated in Mueller–Hinton nutritious media at 37 °C for 24 h, obtaining a bacteria concentration of 5 × 10^5^ colony-forming units (CFU) per mL [53].

The MgO NP minimum inhibitory concentration (MIC) and minimum bactericidal concentration (MBC) were determined by macro- and micro-dilution in Mueller–Hinton broth (MHB) and agar (MHA). The MgO NPs were diluted in sterile DIW to obtain stock solutions ranging from 60 to 8000 ppm that later were diluted 1:1 (*v*/*v* in water) in MHB to the desired concentrations to be tested.

In regard to the macrodilution assays, the bacterial growth samples were prepared by mixing 1 mL of bacterial suspension (5 × 10^5^ CFU/mL) in MHB and 1 mL of the MgO NPs solutions on sterilized glass test tubes and incubated for 24 h at 37 °C. A sterility control without added bacteria and a growth control without MgO NPs added were also prepared. The MIC was determined as the MgO NP concentration in which there were no visible turbidity changes in the growth media. To evaluate the bactericidal efficiency of the MgO NPs, 100 μL of the bacterial growth samples were incubated in Petri dishes with sterile MHA at 37 °C for another 24 h. The MBC was determined to be the concentration at which there was no visible colony growth in the agar.

For the case of the microdilution assays, we used 120 μL of the bacterial suspension (5 × 10^5^ CFU/mL), 120 μL of the MgO NPs solutions, and 10 μL of resazurin (7 mg/mL). The metabolic active bacteria reduced the resazurin into resorufin, turning from a blue to pink color; thus, the non-viable bacterial samples remained blue. *Staphylococcus aureus* samples were placed in the rows A, B, and C of a 96-well plate, while the *Escherichia coli* samples were in the rows D, E, and F. The MgO NPs concentrations of 8000, 4000, 3000, 2000, 1000, 500, 250, 120, 60, and 30 ppm varied from columns 1 to 10, respectively.

On the other hand, ceftriaxone, a third-generation antibiotic, was tested as a positive control at concentrations of 8, 4, 2, 1, 0.5, 0.25, 0.125, 0.06, and 0.03 ppm on columns 1 to 10, for both *S. aureus* and *E. coli*, in rows G and H, respectively. Finally, column 11 was a growth control, and column 12 was tested as a sterility control. The color change was evaluated visually and any change to purple or pink was registered as viable bacteria. The lowest concentration at which a color change was registered was considered the MIC for each bacterium.

Lipidic peroxidation: malondialdehyde (MDA) is a final product of the lipidic peroxidation that was quantified by monitoring the reaction of thiobarbituric acid (TBA) by forming the MDA–TBA adduct that has absorbance in the 530–540 nm wavelength range. Both bacteria were treated for 24 h with the MgO NPs in a 96-well plate, and the MDA was quantified using the TBARS kit by R&D SYSTEMS [23].

## 3. Results and Discussion

### 3.1. MgO Nanoparticles Characterization

**Figure 1:**
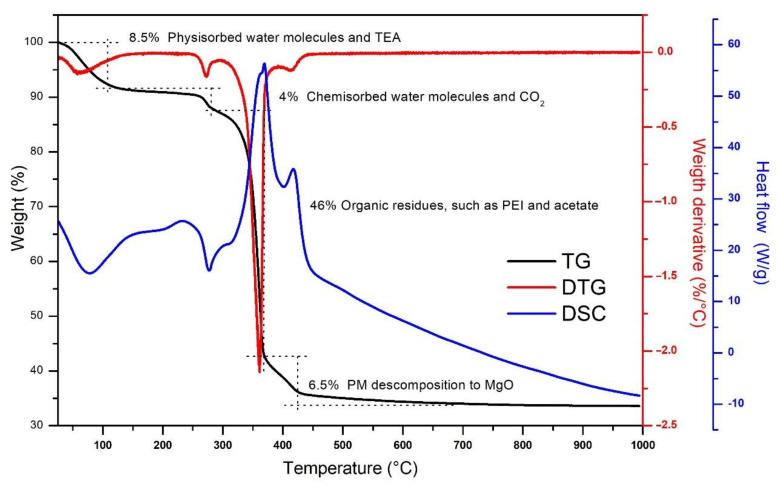
The precursor microparticles (PMs) thermogravimetric curve (TG, black curve), the first derivative of the TG (DTG, red), and the differential scanning calorimetry (DSC, blue) obtained in an extra-dry air atmosphere.

MgO NPs were obtained after the calcination of the PM at 800 °C, as explained in Section 2. The PM was assayed by thermogravimetric analysis (TGA) and differential scanning calorimetry (DSC) techniques (see Figure 1). The TG trace showed a weight loss of 8.5% at 100 °C, associated with the evaporation of the physisorbed water and the TEA residues embedded in the surface of the PM powder [3,54]. From 100 to 280 °C, there was a 4% reduction in weight due to the chemisorbed water molecules evaporation and CO_2_ release [3,55,56].

The most prominent thermal transition was appreciated between 280 and 365 °C, when the mass was reduced 46%; it is in this region of temperatures where the mayor part of the organic residuals can be calcinated and the decomposition of magnesium acetate into MgO occurs—this explains the large weight decrease detected [49,57,58]. This was confirmed by the DSC trace that showed an endothermal maximum in this region. From 365 to 730 °C, the weight loss was only 6.5%; this thermal transition corresponds to the calcination of PEI residuals and the final decomposition of PM to MgO [3,59]. Finally, from 730 to 1000 °C, the DSC indicated that in this temperature range there was a stabilization process of the material and the formation of MgO NPs, but no significant weight loss was detected, and the TG trace stabilized at 33%. For this reason, we assumed that our thermal synthesis was completed after the PMs were calcinated for 5 h at 800 °C.

**Figure 2:**
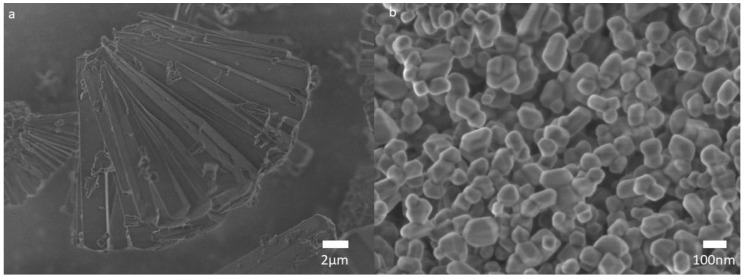
Scanning electron microscopies of (**a**) the precursor microparticles and (**b**) the resulting MgO nanoparticles after the calcination at 800 °C.

Scanning electron microscopy was used to obtain the morphology and particle size. SEM revealed that the PM was mainly composed of elongated crystals of approximately 13 μm in length (see Figure 2a). Figure 2b shows the micrograph of the MgO nanoparticles after the calcination. The sample micrograph depicts a quasi-spherical morphology. The MgO nanoparticles were distributed uniformly with a mean size of 120 ± 42 nm.

**Figure 3:**
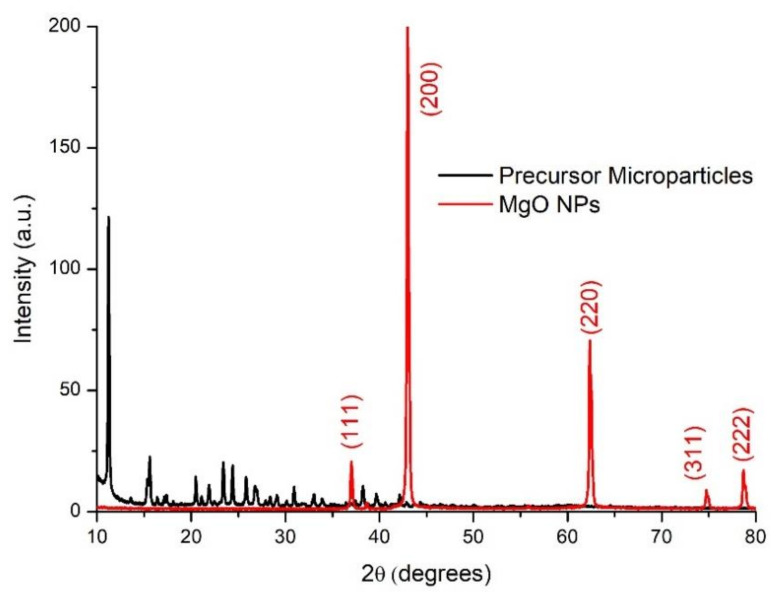
X-ray diffraction patterns for PM and MgO nanoparticles.

Figure 3 shows the X-ray diffraction patterns obtained for the PM and the MgO NPs. The diffraction pattern for the PM shows many peaks in the region from 10 to 40 degrees, indicating that this is a low symmetry crystalline complex. The PM XRD pattern corresponds to anhydrous magnesium acetate, as it was reported earlier [49]. Nevertheless, the calcination process induced a change in the crystalline network, and in the MgO NPs X-ray diffractogram the characteristic peaks can be seen at 37.03, 43.01, 62.37, 74.76, and 78.67 degrees, associated to the Miller indexes (111), (200), (220), (311), and (222), respectively, which corresponds to a face-centered cubic (FCC) network [11]. This result, combined with the TGA, confirmed that the calcination process converted magnesium acetate to magnesium oxide and effectively eliminated the PEI and any organic residues from the PM. The calculated lattice parameter of the FCC MgO cell was 4.209 Å, which was determined according to the procedure described by Askeland [60]. This value is in good agreement with previous reports about MgO nanoparticles with similar size and crystallinity [61].

**Figure 4:**
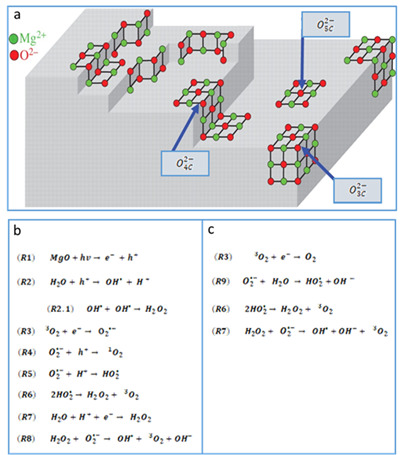
(**a**) Schematic representation of the surface punctual defects on the MgO nanoparticles (NPs). These surface defects (terraces, corners, and steps) provided the sites for low-coordination oxygen ions ($O_{5C}^{2-}$, $O_{4C}^{2-}$ and $O_{3C}^{2-}$, respectively). Reactive oxygen species (ROS) mechanisms under high UV irradiation (**b**) and dark conditions (**c**) (adapted from Chizallet C. et al., 2007 [69]).

The MgO surface was not smooth; therefore, this material presents interesting chemical and optical properties associated with the high concentration of low-coordination oxygen ions ($O^{2-}$_LC_), where there are holes in which electrons can be trapped (Figure 4). These punctual defects can produce a wide variety of highly active chemical species (i.e., $O_{2}$ or ${\mathrm{HO}}^{-}$) by means of thermal, mechanical, and electric processes, but also when the MgO is irradiated with light. When MgO is brought to the nanometric scale, the surface to volume ratio increases, and, with this, punctual defects on the material.

Therefore, there are anions and cations ($O^{2-}$ and ${\mathrm{Mg}}^{2+}$) that can remove the material, and it is in these sites where the electrons can be trapped; these oxygen vacancies trap electrons and form the Faber centers, or color centers (e.g., F, F^+^, F_2_^1+^, F_2_^2+^, etc.) and cationic vacancies (e.g., V_Mg_^0^, V_Mg_^−1^, V_Mg_^−2^), interstitial oxygen, Schottky defects, etc., [62,63,64]. These trapped electrons on the vacancy spaces can absorb light when they are excited by UV photons, as described by the reaction R1 (see Figure 4b). The MgO nanoparticles were analyzed by UV–Vis spectroscopy to determine if there were vacancy sites and, if present, to which type of coordination they corresponded.

**Figure 5:**
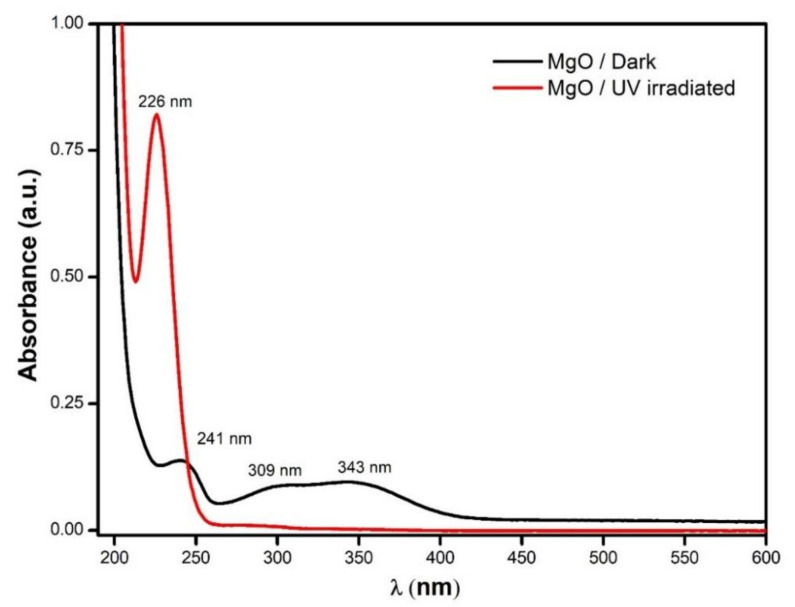
UV–Vis spectra of the MgO nanoparticles when stored in the dark and after irradiation by the UV lamp for 4 h.

The MgO NPs were stored in a light-covered vial and the UV–Vis absorbance spectrum revealed a high absorbance for wavelengths below 190 nm and three peaks at 241, 309, and 343 nm (Figure 5). The absorption data were extrapolated to the Tauc relation: the plot of (*αhν*)^2^ versus the energy of the photons (*hν*), where α is the absorbance value, h is the Plank constant, and ν is the frequency of the photons. When the absorption data are traced by the Tauc relation, it will show one or more straight lines; these lines will intercept with the energy (*hν*) axis, and this intercept gives the value of the energy gap (Eg).

**Figure 6:**
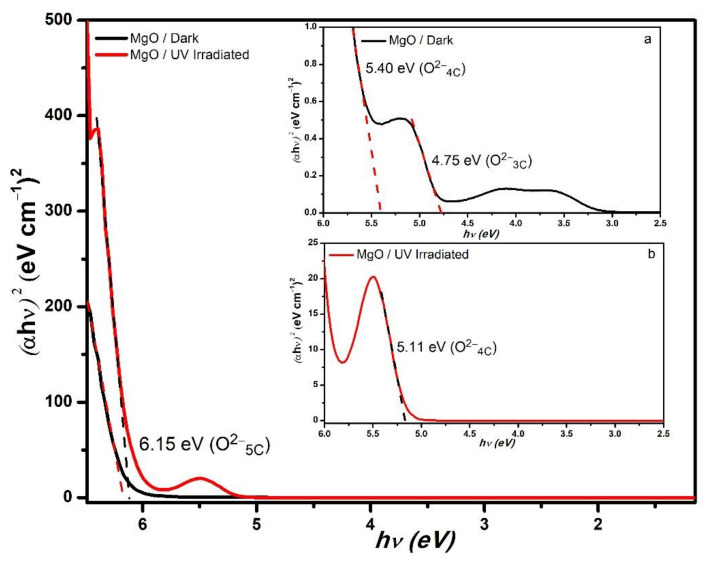
Tauc plots for the MgO nanoparticles stored in the dark and those irradiated by the UV lamp for 4 h. Insets (**a**,**b**) show a zoom to the curves in order to appreciate the low absorbance region.

This Eg for bulk MgO was transparent for photos with energies below 7.8 eV (~160 nm); however, the punctual defects or color centers presented low absorption in the visible part of the spectrum [65]. Thus, it was expected that our MgO NPs presented absorption bands in the visible part of the spectrum because the occurrence of punctual defects was enhanced by the nanometric scale. Figure 6 presents the Tauc plot of the MgO NPs sample, where the intercepts are associated to photons of energies 6.15, 5.40, and 4.75 eV, which corresponds to oxygen low coordination sites associated with terraces ($O_{5C}^{2-}$), corners ($O_{4C}^{2-}$), and steps ($O_{3C}^{2-}$), respectively (see Figure 4a) [19,21,22,66,67,68,69,70].

Additionally, a MgO NP sample was irradiated by intense 365 nm UV light for 4 h; in this case, the UV–Vis absorption spectra also presented high absorption for wavelengths below 200 nm, but contrary to the NP sample kept in the dark, it only presented one intense absorption band at 226 nm (Figure 5). In Figure 6, the Tauc plot showed intercept energies of 6.15 eV (terraces associated with $O_{5C}^{2-}$) and 5.11 eV (corners associated with $O_{4C}^{2-}$) [21,22,34,70,71]. The intense UV radiation enhanced the presences of the Farbe centers associated with the oxygen vacancies of coordination 4C, while the Farbe centers of 3C were diminished; however, to confirm this hypothesis, it would be necessary to perform additional experiments, such as electron spin resonance. We are considering this for our future research.

**Figure 7:**
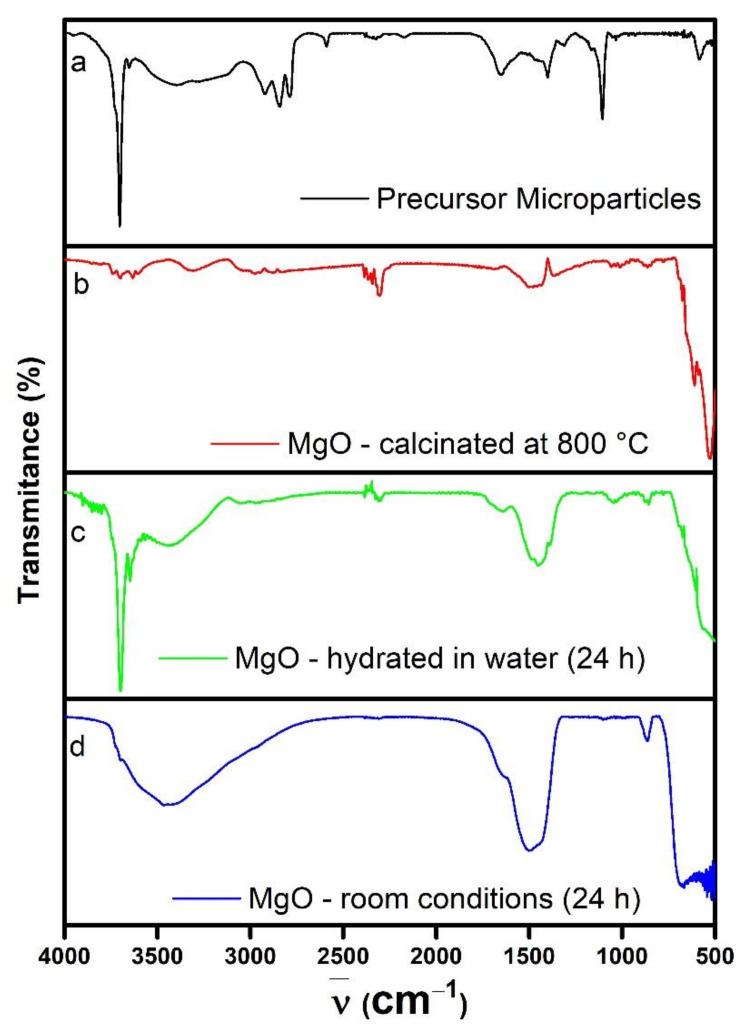
Fourier-transform infrared (FTIR) transmittance spectra of the PM before the calcination (**a**), MgO nanoparticles just after the calcination at 800 °C (**b**), the MgO nanoparticles hydrated in water for 24 h (**c**), and the MgO nanoparticles stored under room conditions for 24 h (**d**).

Figure 7a shows the Fourier-transform infrared spectra (FTIR) obtained for the PM; the obtained signals correspond to those reported for the stabilizing PEI, the TEA base, and magnesium acetate. The sharp and strong peak at 3700 cm^−1^ is attributed to the stretching mode of the OH group [72,73]. There is a band from 3550 symmetric mode to 3120 cm^−1^ asymmetric mode which corresponds to the secondary amines of the PEI, an asymmetric mode of C–H at 2922 cm^−1^ (TEA), two symmetric vibrations of –CH_2_ in PEI at 2843 and 2787 cm^−1^, and two vibration peaks at 1656 and 1107 cm^−1^ associated to the secondary and tertiary amines of the PEI and TEA, respectively. The asymmetric and symmetric stretching for the magnesium acetate appeared at 1589 and 1402 cm^−1^, respectively, and weak bands are also observed at 1313 and 1054 cm^−1^ due to symmetric deformation and rocking CH_3_ mode [74,75,76,77]. Finally, a peak deformation C=O mode occur at 590 cm^−1^, these spectral features indicate that the surface of the magnesium acetate precursor is covered by the PEI and TEA residuals.

On the other hand, Figure 7b shows that, after the calcination process, the MgO nanoparticles had an absorption band 710 to 500 cm^−1^, associated with the Mg–O vibrations in MgO nanoparticles [29,78]. The sharp peak at 3711 cm^−1^ associated with the OH groups present in the PM sample disappeared due to the evaporation of the superficial water molecules during the calcination; the same occurred to the bands and peaks of the PEI and TEA present in the PM spectrum. The water molecule detachment generated basic and acid sites that trapped carbonate ions that were chemisorbed on the MgO surface. The presence of carbonate groups was confirmed by the 1504 cm^−1^ band [44].

The MgO NPs were meant to be used as a water suspension; therefore, it was important to elucidate how they would behave in this chemical environment. For this, 50 mg of the MgO NPs were mixed with 20 mL of water for 24 h; the FTIR spectrum showed that the 3700 cm^−1^ sharp peak, associated to the OH groups that form hydrogen bridges, was present again (Figure 7c). This indicated that water molecules were chemisorbed. Two absorption bands that correspond to the stretching vibrations of the physisorbed water molecules (H–OH) were detected (from 3560 to 3160 cm^−1^ and from 1740 to 1320 cm^−1^) [72,79].

On the other hand, when the MgO NPs were left in an uncovered recipient for 24 h under room conditions (Figure 7d); the spectra showed broad absorption bands (from 3760 to 2750 cm^−1^ and from 1780 to 1330 cm^−1^), which indicates that the MgO NPs were only hydrated by physisorbed water molecules, due to the inherent hygroscopic properties of the material. The FTIR spectrum showed the presence of chemisorbed and physisorbed species on the surface of the MgO NPs, such as those shown in Figure 7.

### 3.2. Reactive Oxygen Species Production by the MgO NPs

**Figure 8:**
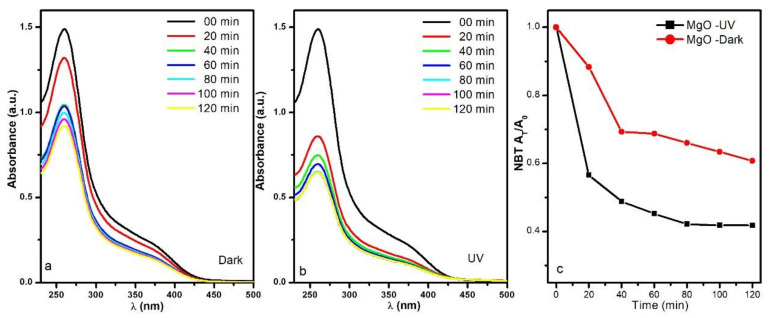
UV–Vis spectra of nitro blue tetrazolium (NBT) in nano MgO under (**a**) dark and (**b**) UV irradiation, and (**c**) the degradation kinetics of NBT in the dark and with UV irradiation.

In addition to the interesting physical and chemical properties that the MgO NPs have shown, it was also important to elucidate if the MgO NPs can produce reactive oxygen species (ROS) such as superoxide anion radicals or hydrogen peroxide ($H_{2}O_{2}$), because these ROS are related to the antimicrobial effect of this nanomaterials. For this, the nitro blue tetrazolium assay was performed to monitor the production of superoxide anion radical by analyzing the degradation of NBT by UV–Vis absorbance spectroscopy (see Figure 8).

For the sample maintained at 43 °C and under dark conditions, the NBT absorption at 259 nm diminished from 1.50 to 1.03 in the first 40 min, followed by a slower $O_{2}^{•-}$ production, and at the end of the 120 min assay, the NBT absorption band reduced to 0.92 (Figure 8a). On the other hand, when the MgO NPs were irradiated by the UV lamp during the experiment, the NBT absorbance was reduced dramatically faster; it went from 1.50 to 0.86 in just 20 min and stabilized around 0.64 in 80 min (Figure 8b).

Because the absorbance is directly related to the concentration of NBT, we can estimate the superoxide anion radical production; these results are shown in Figure 8c. The NBT concentrations were reduced to 60% and 42% for the UV irradiated sample and the one kept in the dark, respectively. Therefore, the results obtained for superoxide production were 50.22 mg/g for the sample kept in the dark and 74.51 mg/g for the UV irradiated sample. The superoxide production was enhanced by the UV illumination, but even in dark conditions, the MgO NPs could produce this reactive oxygen species.

**Figure 9:**
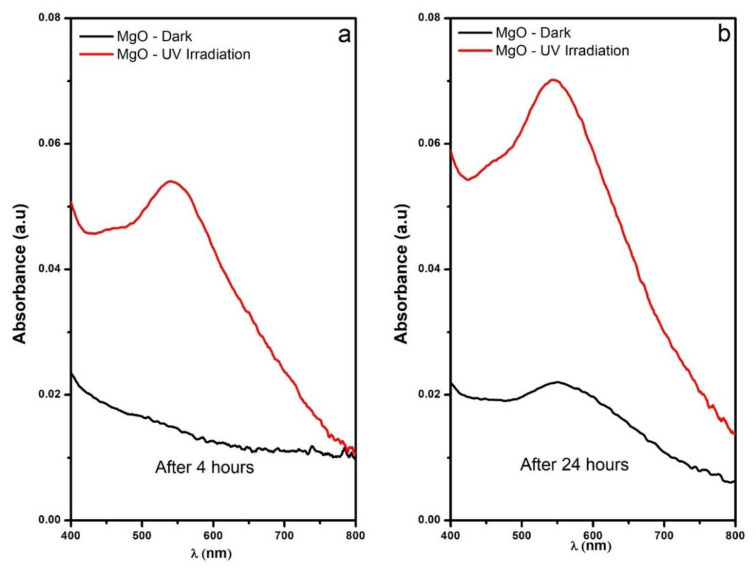
UV–Vis absorbance spectra of the iodine–starch complex used to quantify the $H_{2}O_{2}$ production by the MgO NPs under irradiation UV and dark conditions; (**a**) 4 h and (**b**) 24 h.

A calibration curve using the iodometry technique was constructed so that the $H_{2}O_{2}$ productions could be calculated directly from the UV–Vis data. The MgO NPs were tested under two different illumination conditions (intense UV lighting and dark conditions) for 24 h. Figure 9 shows the spectra recorded at 4 and 24 h; during the first 4 h, the 545 nm band increased considerably for the sample irradiated with the UV lamp, while for the sample kept in the dark, this was barely detected.

After 24 h under the previously mentioned conditions, the spectral changes were more evident for the two samples and the $H_{2}O_{2}$ productions were calculated to be 1.50 μg/g for the UV irradiated sample and 0.46 μg/g for the sample kept in the dark. Just like in the $O^{2-}$ assay, the production of $H_{2}O_{2}$ was enhanced by the UV illumination, but again, the MgO NPs were able to produce under dark conditions. The MgO NPs showed the capacity for ROS production under UV illumination and dark conditions; therefore, the mechanisms shown in Figure 4b,c are proposed.

When the MgO NPs suspensions were irradiated by UV photons (Figure 4b), charge carriers were produced, as expressed in the reaction R1. The holes can react with the water molecules absorbed in the NP surface, forming highly reactive hydroxyl radicals that can react with other ${\mathrm{OH}}^{•}$ to form $H_{2}O_{2}$ (reactions R2 and R2.1). The dissolved triplet oxygen (^3^O_2_) acts as an electron acceptor, forming a superoxide anion radical ($O_{2}^{•-}$) (R3).

According to the FTIR, these NPs have a high presence of catalytic sites that can decompose ^3^O_2_ into superoxide anion radicals by the reduction of a single electron. Given that the NP surfaces are covered by a basic environment (${\mathrm{HO}}^{-}$), the superoxide species are stable and exist in high concentrations in the NP surfaces and the water suspension [66]. These superoxide anion radicals can react with holes in the NP surface to regenerate the reaction cascade through the singlet oxygen (^1^O_2_) (see reaction R4), or continue like an oxidizing agent but also as a source of hydroxyl radicals for the formation of $H_{2}O_{2}$, as expressed in reactions R5 to R7. Finally, the $H_{2}O_{2}$ can be decomposed in ${\mathrm{OH}}^{•}$, ^3^O_2_, and ${\mathrm{OH}}^{-}$, as shown in reaction R8.

The Tauc analysis confirmed the presence of point defects on the surface of the MgO NPs; these sites are occupied by an electron that can react with the dissolved oxygen triplet (^3^O_2_) to form the radical superoxide anion ($O_{2}^{•-}$), as expressed in reaction R3. The superoxide anion radical can decompose water molecules into hydroperoxyl radicals (${\mathrm{HO}}_{2}^{•}$) and hydroxide ions (${\mathrm{OH}}^{-}$) (R9), which gives way to reactions R6 and R7, which will continue the cycle, as shown in Figure 4c, both in the presence of light and in darkness.

### 3.3. MgO NPs Antibacterial Effect Evaluation

MgO was proven to produce ROS such as $O_{2}^{•-}$ and $H_{2}O_{2}$; therefore, we decided to evaluate the antibacterial properties using two bacterial strains: *Escherichia coli* and *Staphylococcus aureus*. These bacteria were tested in two ways: in macrodilution conditions, the antimicrobial effect was evaluated visually in broth and agar media; on the other hand, in microdilution conditions, the resazurin assay was performed to discriminate between the viable and non-viable bacteria cultures.

**Figure 10:**
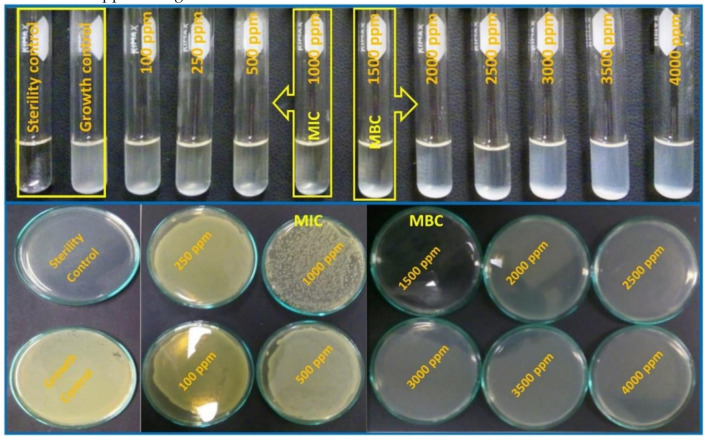
*Escherichia coli* ATCC 25,922 exposed to different concentrations of MgO NPs. The test tubes’ turbidity from 3000 to 4000 ppm was associated with the scattering of MgO NPs. On the upper photographs, the Petri dishes show that for concentrations of 500 ppm and below, the growth was the same as in the growth control. MIC: 1000 ppm; MBC: 1500 ppm.

*Escherichia coli*, a Gram-negative type of bacteria, was exposed for 24 h to MgO NP concentrations ranging from 100 to 4000 ppm. Visually, the turbidity, associated with the bacterial growth in the MHB, was considerably reduced for the sample prepared with 1000 ppm of MgO NPs. For this reason, we determined that this was the MIC (see Figure 10). This result was confirmed when 100 μL of the bacterial growth samples were incubated in sterile MHA for 24 h; the samples that were exposed to 100, 250, and 500 ppm presented the same bacterial growth as the control, whilst the bacterial growth of the 1000 ppm sample was considerably reduced. On the other hand, for samples of concentrations 1500 ppm and higher, there was no bacterial growth in the Petri dishes; hence, we concluded that the MBC was 1500 ppm of MgO NPs.

**Figure 11:**
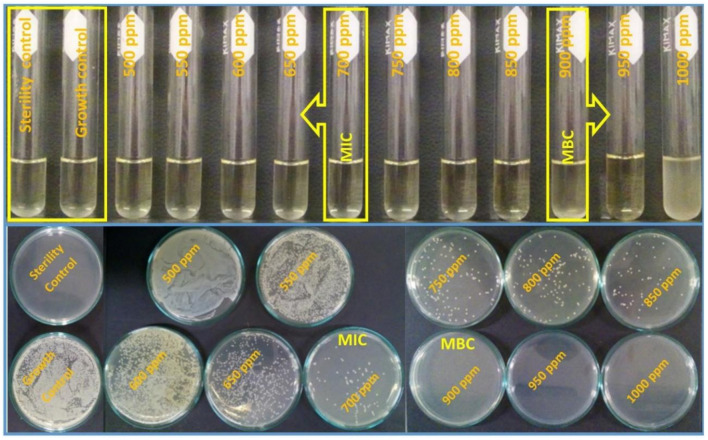
*Staphylococcus aureus* ATCC 25,923 exposed to different concentrations of MgO NPs. The upper photographs show that for concentrations of 650 ppm and below, there were many colony-forming units (CFU). MIC: 700 ppm; MBC: 900 ppm.

The macrodilution experiments were performed for *Staphylococcus aureus* (Gram-positive bacteria); however, in this case, the MgO NPs concentrations tested varied from 500 to 1000 ppm. It was not possible to determine the MIC and MBC from the bacterial suspension samples, but once the broths were incubated in the agars for 24 h, it was easy to perceive the differences among the samples (Figure 11). The MIC was determined to be 700 ppm of MgO NPs, because the number of colonies formed was visibly lower than in the samples with lower concentrations. On the other hand, the MBC corresponded to 900 ppm of MgO NPs, where no bacteria colonies grew in the agar.

**Figure 12:**
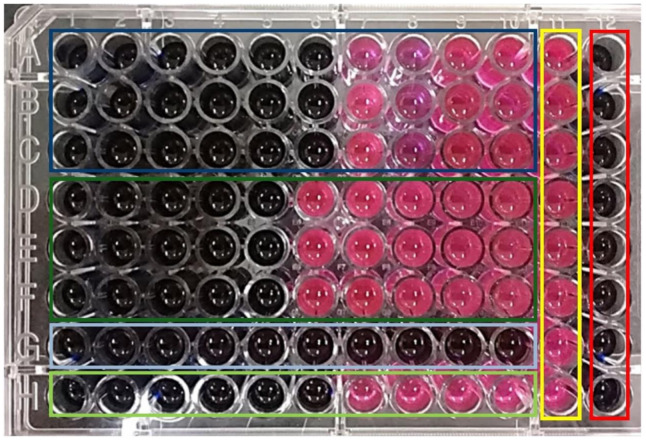
The 96-well plate used in the microdilution assay: the MgO NPs and ceftriaxone antibiotic concentrations decrease from left to right in rows 1 to 10 (8000, 4000, 3000, 2000, 1000, 500, 250, 120, 60, and 30 ppm of MgO NPs). The wells inside the dark blue rectangle correspond to the *S. aureus* exposed to the MgO NPs, whilst the light blue correspond to the same bacteria exposed to ceftriaxone. The wells inside the dark and light green rectangles correspond to the *E. coli* exposed to the MgO NPs and the antibiotic, respectively. The yellow rectangle was the growth control, and the red rectangle was the sterility control.

The resazurin assay was carried out in microdilution conditions, as a method of evaluating the antibacterial effect of the MgO NPs (Figure 12). Our results indicated that, under these conditions, the MIC for *E. coli* was 500 ppm of MgO NPs, which was the highest concentration that showed the color change from blue to pink (dark green rectangle in Figure 12), while for the *S. aureus,* the MIC was 250 ppm of MgO NPs (dark blue rectangle); this indicates that *S. aureus* was more susceptible to the antimicrobial effects of the MgO NPs than the Gram-negative bacteria *E. coli*.

The bacterial membrane plays a crucial role in the behavior of the diverse bacterial strains because, in this site, there are a great variety of macromolecules that regulate many biological functions, such as the protection of the microbe, absorption of nutrients, and the expulsion of waste substances. This membrane is chemically multifunctional; therefore, it can interact with many compounds, including our MgO nanomaterial.

The bacterial resistance to antibiotic drugs or materials has morphological and functional origins. In general, the Gram-negative bacteria have greater resistance at a morphological level due to the outer cell membrane they present, which is absent in Gram-positive bacteria [80]. At a functional level, said outer cell membrane has anchored proteins called porins; these proteins function as a filter allowing or prohibiting the entry of material into the bacteria, which determines the membrane’s permeability. In the presence of antibiotics, porins strongly influence bacterial resistance [81,82]. On the other hand, the layer of polysaccharides presents in Gram-negative bacteria, which surrounds the outer cell membrane, protects the bacteria against lipid peroxidation and consequently reduces the antimicrobial action [83]. Therefore, the morphological and functional differences between Gram-positive and Gram-negative bacteria were reflected in our results. Although MgO NPs adhere to both bacteria’s cell membranes, the morphological structure of *E. coli* as a Gram-negative bacterium allows presenting less sensitivity to the action of MgO NPs compared to *S. aureus*, a Gram-positive bacterium.

**Figure 13:**
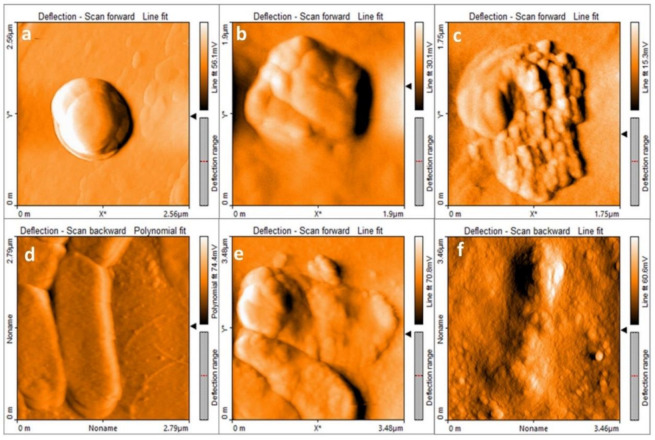
Atomic force microscopy (AFM) images obtained for the non-exposed *S. aureus* (panel **a**) and those treated for 24 h with 250 (MIC) and 500 ppm MgO NPs ((**b**) and (**c**), respectively). The non-treated *E. coli* is shown in panel (**d**), while panels (**e**,**f**) show those treated with 500 (MIC) and 1000 ppm MgO NPs, respectively.

Atomic force microscopy (AFM) was used to observe the morpho-structural changes the MgO NPs induced in the bacterial strains. The non-treated *S. aureus* bacteria showed a rounded shape with a mean diameter of 1 ± 0.15 μm and a mean height of 200 ± 50 nm (see Figure 13a). On the other hand, when *S. aureus* was treated for 24 h with MgO NPs at the MIC (250 ppm), as showed in Figure 13b, the shape of the bacteria was no longer round; the surface was corrugated, and the mean height was diminished by 40% (120 ± 5 nm).

Figure 13c shows the effect of treating *S. aureus* at 500 ppm; in this case, the bacterial membrane was severely damaged, and the cytosol material was exposed. Similar morphological changes were detected for the *E. coli* strain; the non-treated bacteria presented a bacillus shape and a smooth surface with a mean height of 160 ± 50 nm (Figure 13d). Most of the bacteria treated with 500 ppm (MIC) presented a rough surface, and the mean height was diminished to 76 ± 10 nm (Figure 13e), while in the bacteria treated with 1000 ppm MgO NPs, the structures were collapsed, and the cytosol was exposed (Figure 13f).

This indicates that over the MICs, both bacteria, *E. coli* and *S. aureus*, were severely damaged and unviable, which can be explained by the lipidic peroxidation results. The TBARS assay showed that the MgO NPs increased the MDA in 28% for the case of *S. aureus* treated with 500 ppm and 25% for the of *E. coli* treated with 1000 ppm when compared with the non-treated control bacteria. These results are similar to those reported by Krishnamoorthy et al. [21,22].

According to the XRD and UV–Vis results, our MgO NPs presented an FCC crystalline network composed of Mg^2+^ and O^2−^ units; however, on the surface of the NPs there were punctual defects that gave sites for low coordination oxygen ions (O^2−^_LC_). The FTIR spectra showed that these sites also facilitated the proton (H^+^) recombination to form hydroxyl groups in various environments (aqueous and gaseous), giving the material an alkaline character, which is one the main factors in its antibacterial properties. The O^2−^ ions and the punctual surface defects are the principal factors that allow the generation of ROS, such as $O_{2}^{•-}$ and ^1^O_2_, triggering multiple reactions, including the formation of $H_{2}O_{2}$, and continuing the redox cycle previously proposed (Figure 4b,c).

In this work, the radical anion superoxide and the non-radical $H_{2}O_{2}$ species are highlighted. The presence of superoxide ($O_{2}^{•-})$ was confirmed by the NBT assay. This radical is very stable under alkaline conditions and can interact with carbon atoms with electron deficiencies, such as the C=O groups in the peptide macromolecules in the membrane or inside the bacteria. The lipidic peroxidation results confirm the formation of the MDA–TBA adduct as a subproduct of this uncontrolled reaction promoted by the MgO NPs.

As proposed before, an excess of superoxide anion radical interacted with H^+^ to form $H_{2}O_{2}$, whose production was confirmed by the respective assay. This non-radical species is permeable and soluble among the lipids and can permeate the bacterial membrane, starting the redox of some of the important structures that maintain the homeostasis and, hence, the viability. The formation of the MDA–TBA adduct and the Resazurin assay results provide the guideline that the $H_{2}O_{2}$ is the main cause of the lipid fragmentation, causing the instability observed by the AFM images, where the membrane degradation and the cytosol exposure can be seen (Figure 13).

## 4. Conclusions

In this work, we presented a novel MgO NPs synthesis method using an uncommon precipitant, triethylamine, during the solvothermal synthesis. The MgO NPs were characterized, and their physical and chemical properties were obtained. The characterization results indicate that our material attacked and demonstrated an antibacterial effect against Gram-negative and Gram-positive strains under illumination and under dark conditions.

The antibacterial activity of the MgO NPs evaluated in *E. coli* and *S. aureus* bacteria was attributed to the basic environment these particles generate and to the generation of superoxide anion radicals and hydrogen peroxide that feeds the redox cycle, which we also proposed. This work supports the idea that the MgO toxicity is attributed to the presence of superficial punctual defects and oxygen vacancies, which allows the generation of ROS that can degrade the lipids in the bacterial membranes as well as affect the biochemical processes inside the microorganisms.

AFM images confirmed the lipidic peroxidation results and allowed us to observe the different degrees of damage caused to both types of bacteria at the MIC and higher NP concentrations, and we were even able to appreciate the cytosol exposure when the bacterial structure collapsed. In summary, we have proposed a method for the synthesis of nanoparticles through triethylamine-based precipitation to obtain MgO NPs that can be used as broad-spectrum antibacterial agents.

## Data Availability

The data presented in this study are available on request from the corresponding author.
